# Long-term results of topical 0.02% tacrolimus ointment for refractory ocular surface inflammation in pediatric patients

**DOI:** 10.1186/s12886-021-01998-0

**Published:** 2021-06-05

**Authors:** Kyungmin Koh, Ikhyun Jun, Tae-im Kim, Eung Kweon Kim, Kyoung Yul Seo

**Affiliations:** 1 Kim’s Eye Hospital, Seoul, Republic of Korea; 2 Department of Ophthalmology, Institute of Vision Research, Yonsei University College of Medicine, Seoul, Republic of Korea; 3 Saevit Eye Hospital, Goyang, Republic of Korea

**Keywords:** Pediatric patient, Refractory ocular surface inflammation, Topical tacrolimus

## Abstract

**Background:**

No studies have been reported on the efficacy and safety of long-term (≥12 months) use of topical tacrolimus for refractory ocular surface inflammation in pediatric patients.

**Methods:**

Medical records of pediatric patients who were prescribed topical 0.02% tacrolimus ointment for refractory ocular surface inflammation between January of 2010 and March of 2018 were reviewed retrospectively. Changes in ocular surface signs during slit-lamp examination, clinical symptoms and concurrent steroid use were graded with a scoring system. The presence of side effects was also assessed. The changes in disease severity and patient symptoms were compared between baseline and after the treatment.

**Results:**

Among 72 patients (55% males, mean age 10.8 ± 3.9 years, range 3 to 17 years), 25 patients (48% males, mean age 11.4 ± 3.9 years) fully recovered, resulting in discontinuance of the ointment treatment before 12 months. Six patients experienced intolerable burning sensation, which required treatment cessation. Cessation days of those who quit were 1,5,14,20,26, and 35 days. Seven patients were lost during follow-up. Thirty-four patients (56% males, mean age 11.2 ± 4.2 years, range 3 to 17 years) were treated with tacrolimus ointment for over 12 months (average 23.1 ± 19.1 months, range 12 to 98 months). During the follow-up period, all patients showed improved clinical signs and symptoms, and no adverse reaction was noted.

**Conclusions:**

Long-term maintenance of topical tacrolimus 0.02% ointment is safe and effective in improving refractory ocular surface inflammation in pediatric patients.

**Supplementary Information:**

The online version contains supplementary material available at 10.1186/s12886-021-01998-0.

## Background

The ocular surface inflammation management requires intense immunosuppression [1]. T helper 2 cells play a vital role in the pathogenesis of vernal keratoconjunctivitis (VKC) [2]. In atopic keratoconjunctivitis (AKC), both T helper 1 and 2 cytokines are expressed in the irritated conjunctiva [3]. Chronic ocular Graft-versus-host disease (GVHD) occurs by reactive T cell [4]. In ocular cicatricial pemphigoid (OCP), T cells are responsible for producing conjunctival scarring [1]. Stevens–Johnson syndrome (SJS) lesions are produced by the migration of cytotoxic T lymphocytes [5]. The pathogenesis of phlyctenular keratoconjunctivitis (PKC) is delayed-type hypersensitivity [6].

Topical steroids are the main treatment for these diseases. However, prolonged steroid use can potentially cause severe adverse reactions, including steroid-induced glaucoma (SIG), posterior subcapsular cataract, and secondary infection [7]. Pediatric patients tend to show a more severe response to topical steroids compared to adults [7, 8]. In one study involving 1259 children with glaucoma, 4.7% were cases of SIG. Of these patients, 87% had been prescribed with topical steroids for VKC [7].

To overcome the limitations of steroids, topical immunosuppressants have been used as an alternative. Tacrolimus is a nonsteroidal macrolide immunosuppressant isolated from *Streptomyces tsukubaensis* and is known to be 30 ~ 100 times more powerful than cyclosporine [9]. The mechanism by which tacrolimus suppresses inflammatory reactions is not clear. So far, it has been discovered that tacrolimus attaches to FK506-binding proteins within T lymphocytes and suppresses calcineurin activity [10]. Subsequent inhibition of T lymphocytes results in the inhibition of release of inflammatory cytokines [1], including IL-2 from T lymphocytes [11]. The application of topical tacrolimus is effective in treating various T-cell-mediated ocular diseases [1].

Many studies have described satisfactory results with topical tacrolimus on various ocular surface inflammation [1, 12, 13]. However, no studies till date have investigated the safety of long-term (≥12 months) use of topical tacrolimus in pediatric patients for treating ocular surface inflammation.

The goal of this paper was to evaluate the efficacy and safety of long-term treatment using topical 0.02% tacrolimus ointment in pediatric patients with ocular surface inflammation refractory to conventional therapy.

## Methods

We retrospectively reviewed the medical records of 72 consecutive patients diagnosed with ocular surface inflammation such as ocular GVHD, VKC, AKC, OCP, SJS, and PKC under 18 years old. The patients had been prescribed with topical 0.02% tacrolimus ointment for refractory ocular surface inflammation between January of 2010 and March of 2018 at the Department of Ophthalmology, Severance Hospital, Yonsei University, Seoul, Republic of Korea from January 2010 to March 2018.

This study was conducted at the Department of Ophthalmology, Severance Hospital, Yonsei University, Seoul, Republic of Korea. The study protocol was approved by the Institutional Review Board of Yonsei University, Seoul, Republic of Korea (IRB number: 4–2019-1315), and it adhered to the tenets of the Declaration of Helsinki. The written informed consent was waived because of the retrospective design and the use of deidentified patient data.

We defined those cases that showed persistence of symptoms and signs despite receiving a topical 0.12 or 1% prednisone acetate or use of systemic steroids treatment for more than 3 months, cases in which there was a relapse after tapering or withdrawal of steroids, and cases in which steroid-related complications developed as “refractory to conventional treatment”. The “refractory to conventional treatment” patients were prescribed with topical 0.02% tacrolimus ointment as an adjunct immunosuppressive therapy in addition to previous steroid treatments [12].

The exclusion criteria were as follows: patients with history of tacrolimus eye drops, ocular bacterial infection, ocular herpetic infection, ocular chemical injury, previous ocular trauma, and contact lens wearer. The following information at the beginning and each follow-up (every month up to 3 months, and every 3 months after that) was obtained from a retrospective chart review: demographic data including diagnosis, systemic disease, clinical features, uncorrected visual acuity (UCVA), best corrected visual acuity (BCVA), intraocular pressure (IOP), disease severity (symptom and sign score), steroid score, and presence of any adverse reaction. Disease severity was classified as absent, mild, moderate, or severe according to a grading system out of four points (0 to 3 points) based on symptoms and signs (Table 1) [14]. Clinical outcomes were assessed using the same grading system. The primary measure of treatment efficacy was a decrease the composite score of symptoms and signs. The changes in the composite score of symptoms and signs were compared between baseline and after the treatment. The slit-lamp examinations and a questionnaire to subject symptoms were conducted by a single clinician (KYS) for consistency. Pediatric patients usually do not allow good slit lamp examination. In case of poor cooperation of the patients, slit lamp examination was performed using portable slit lamp.

**Table 1:**
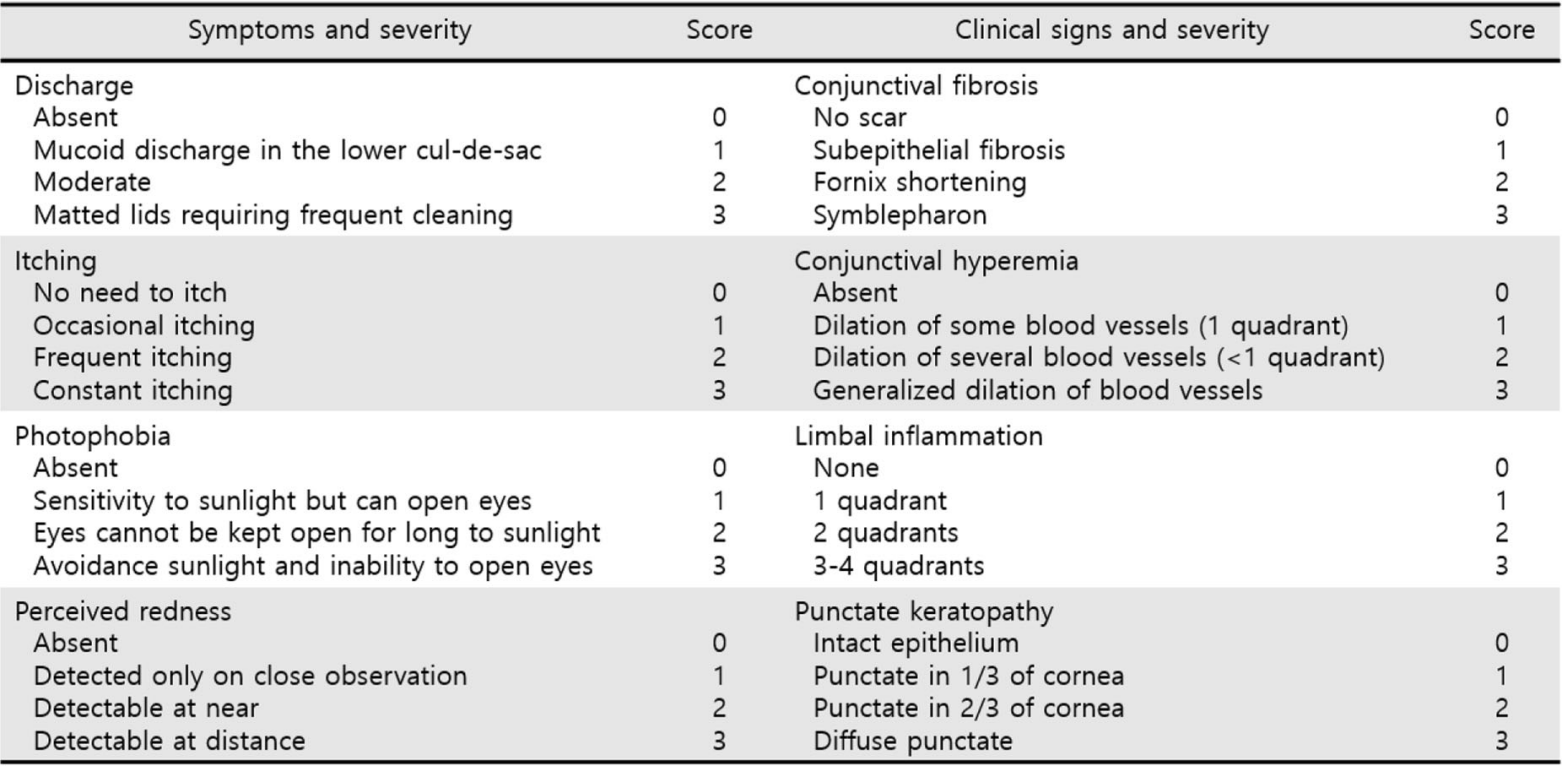
Grading system of disease severity

The use of steroid was categorized and scored on a scale of 0 to 4. The score of 0 indicates no steroid necessary; 1 indicates the use of 0.1% topical fluorometholone (Ocumetholone®; Samil Pharmaceutical Co., Ltd., Seoul, Republic of Korea); 2 indicates the use of 0.12% topical prednisone acetate (Optilon®; Chong Kun Dang Pharmaceutical Co., Seoul, Republic of Korea); 3 indicates the use of 1% topical prednisone acetate (Predforte®; Allergan Inc., Irvine, CA, USA); and 4 indicates the use of systemic steroids with or without concurrent topical prednisone acetate of 1% [12, 15].

The commercial tacrolimus ointment (Protopic® ointment 0.03%; Astellas Pharma, Tokyo, Japan), which has been used to treat dermatologic disorders, is extremely viscous and inappropriate for direct application into the conjunctival sac [4, 12]. Hence, we made a preparation of 0.02% tacrolimus ointment by diluting the 0.03% tacrolimus ointment ratio of 2:1 by volume with a less viscous ophthalmic ointment (Duratears®; Alcon Laboratories, Inc., Fort Worth, Texas, USA). The Duratears® ointment is composed of 30 mg anhydros liquid lanolin per gram of mineral oil base [16]. The patients were instructed to put the topical 0.02% tacrolimus ointment about the size of a rice grain into the conjunctival sac twice a day [4, 12].

All continuous data are expressed as mean ± standard deviation (SD) while categorical data were presented as number and percentage of the total population. Statistical analyses were performed with the SPSS statistical software package (version 20.0; SPSS Inc., Chicago, Illinois, USA). A *p*-value less than 0.05 was considered statistically significant.

## Results

**Fig. 1:**
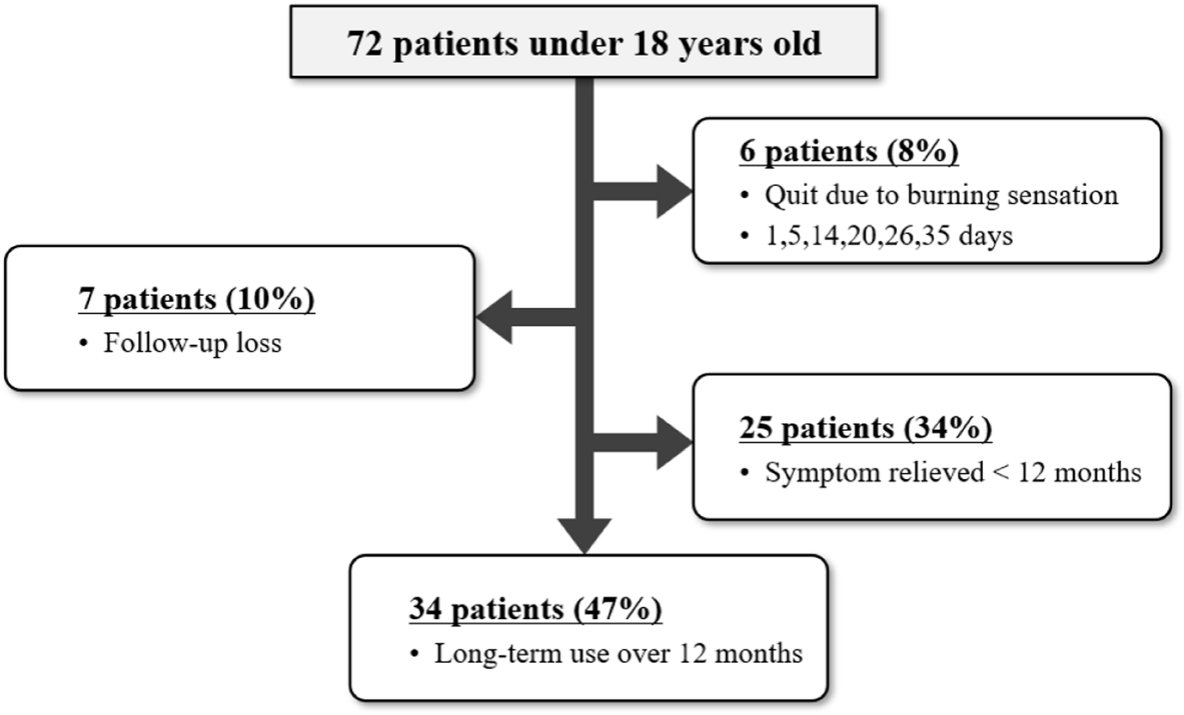
The flowchart of pediatric patients who were treated with the topical 0.02% tacrolimus ointment for refractory ocular surface inflammation

There were 72 patients (55% males, mean age 10.8 ± 3.9 years, range 3 to 17 years) who were prescribed topical 0.02% tacrolimus ointment for refractory ocular surface inflammation between January of 2010 and March of 2018. All patients had bilateral ocular involvement. Seven patients were lost during follow-up. Six patients (17% males, mean age 10.3 ± 3.1 years, range 6 to 14 years) experienced painful burning sensation and withdrew from the tacrolimus treatment. The number of days before the cessation of treatment due to severe burning sensation was 1, 5, 14, 20, 26, and 35 days (Fig. 1). Twenty-five patients (48% males, mean age 11.4 ± 3.9 years, range 4 to 16 years) fully recovered, resulting in discontinuation of the tacrolimus ointment before 12 months. The mean duration of using the tacrolimus ointment in this group was 3.61 ± 2.45 months (range 1 to 8 months). Thirty-four patients (56% males, mean age 11.2 ± 4.2 years, range 3 to 17 years) were treated with tacrolimus ointment for 12 months or more (mean follow-up period 23.12 ± 19.07 months, range 12 to 98 months).

There were no corneal deposits, subepithelial keratitis, ocular surface staining, IOP elevation, infections, or other unfavorable influences associated with the use of topical tacrolimus ointment. No side effects other than burning sensation were identified during the follow-up period.

The distribution of diagnosis of 65 patients (seven patients lost to follow-up were excluded) is as follows: The most common diagnosis was AKC (46%), followed by VKC (35%), GVHD (15%) and PKC (3%). SJS and OCP each accounted for 1%. The mean duration of using the tacrolimus ointment in AKC group (54% males, mean age 10.6 ± 3.8 years, range 4 to 17 years) was 8.89 ± 5.14 months (range 1 to 21 months). Three of 34 patients experienced painful burning sensation which required treatment cessation. The mean duration of using the tacrolimus ointment in VKC group (60% males, mean age 11.5 ± 3.6 years, range 6 to 17 years) was 10.25 ± 3.45 months (range 1 to 20 months). Three of 32 patients experienced painful burning sensation and the treatment was withheld. The mean duration of using the tacrolimus ointment in ocular GVHD group (56% males, mean age 9.24 ± 5.5 years, range 3 to 16 years) was 25.01 ± 14.35 months (range 13 to 55 months). All of PKC (67% males, mean age 13.6 ± 1.5 years, range 12 to 15 years), OCP (female, age 11 years), and SJS (male, age 14 years) patients used tacrolimus ointment for more than 12 consecutive months without reporting any side effects.

**Fig. 2:**
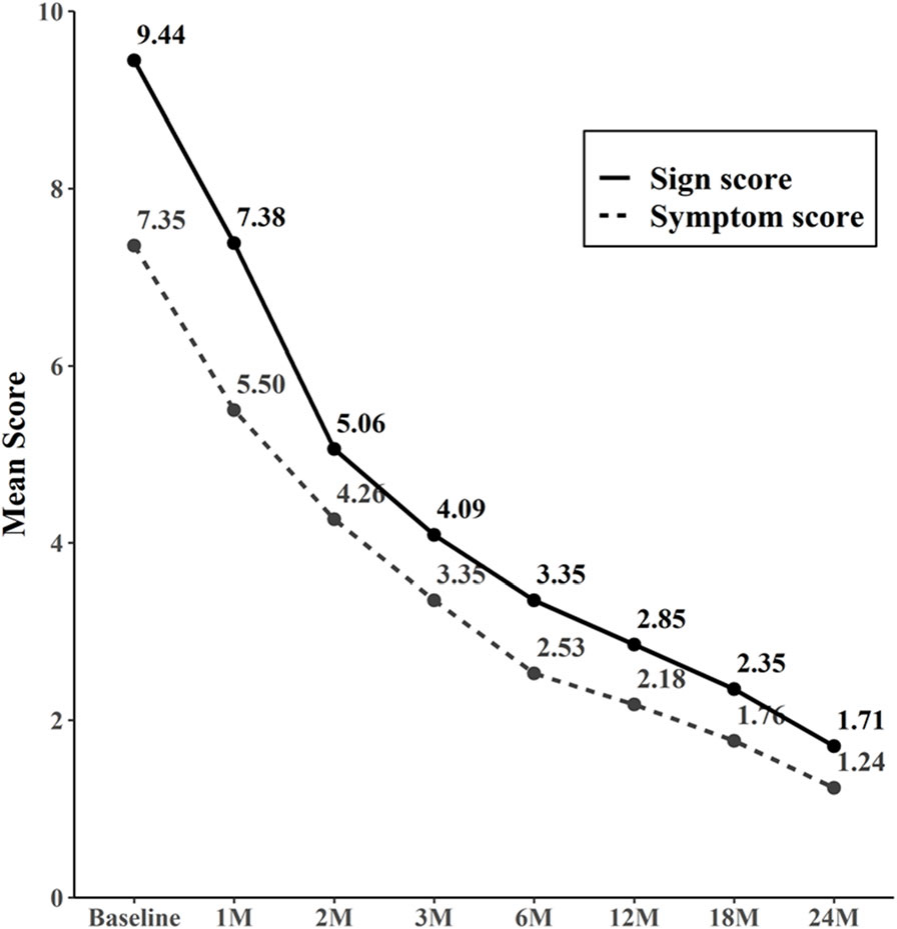
Comparison of overall symptom and sign score during the follow-up period. Both scores decrease during the treatment period

**Fig. 3:**
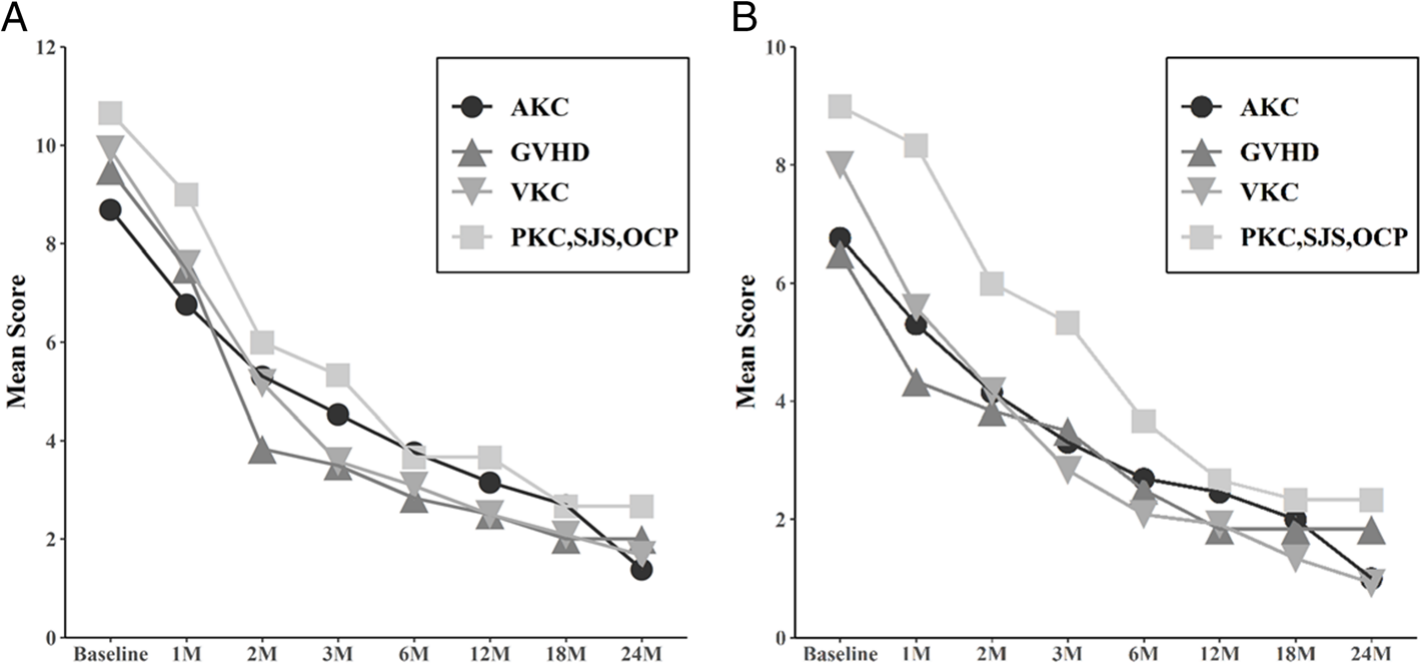
Comparison of each sign scores (**a**) and symptom scores (**b**) among disease groups during the follow-up period. It shows the changes in both scores during the treatment period for each disease

Comparisons of ocular examination results between before and after the treatment of 34 patients with a follow-up period of 12 months or longer showed no significant difference in the UCVA, BCVA, and mean IOP (Table 2). Disease severity was calculated by the sum of symptom and sign scores (Table 1). The composite score of symptoms and signs was computed at the beginning and each follow-up. The mean composite sign score at initial visit was 9.44 ± 2.11 and dropped to 2.85 ± 1.37 at 12 months (*P* < .001) (Fig. 2). The mean composite symptom score at initial visit was 7.35 ± 1.85 and dropped to 2.18 ± 1.08 at 12 months (*P* < .001) (Fig. 2). The changes in mean scores for the symptoms and signs during follow-up are demonstrated in Fig. 2. After 1 month of treatment, significant improvement in symptoms and signs was noted (Fig. 2). The total sign score (range, 0 to 12) significantly decreased 1 month after initiation of topical tacrolimus ointment in all disease groups (Fig. 3a). The total symptom score (range, 0 to 12) also showed a significant decrease from baseline 4 weeks after initiation of topical tacrolimus ointment in all disease groups (Fig. 3b).

**Table 2:** Before and after treatment comparison of parameters associated with therapeutic effects

Variable	Before treatment	Final follow-up	*P*-value
UCVA (logMAR, mean ± SD)	0.44 ± 0.19	0.39 ± 0.13	0.352^a^
BCVA (logMAR, mean ± SD)	0.22 ± 0.31	0.18 ± 0.30	0.199^a^
IOP, mmHg (mean ± SD)	14.82 ± 3.63	15.26 ± 2.57	0.419^a^
Symptom Score	7.35 ± 1.85	1.23 ± 0.95	0.001^b^*
Sign Score	9.44 ± 2.11	1.71 ± 1.06	0.001^b^*
Steroid Score	3.32 ± 0.84	0.58 ± 0.65	0.001^b^*

*UCVA* uncorrected visual acuity, *BCVA* best corrected visual acuity, *logMAR* logarithm of the minimum angle of resolution, *SD* standard deviation, *IOP* intraocular pressure

^a^Paired-t test; ^b^Wilcoxon signed rank test

*P < 0.05 was considered statistically significant

**Fig. 4:**
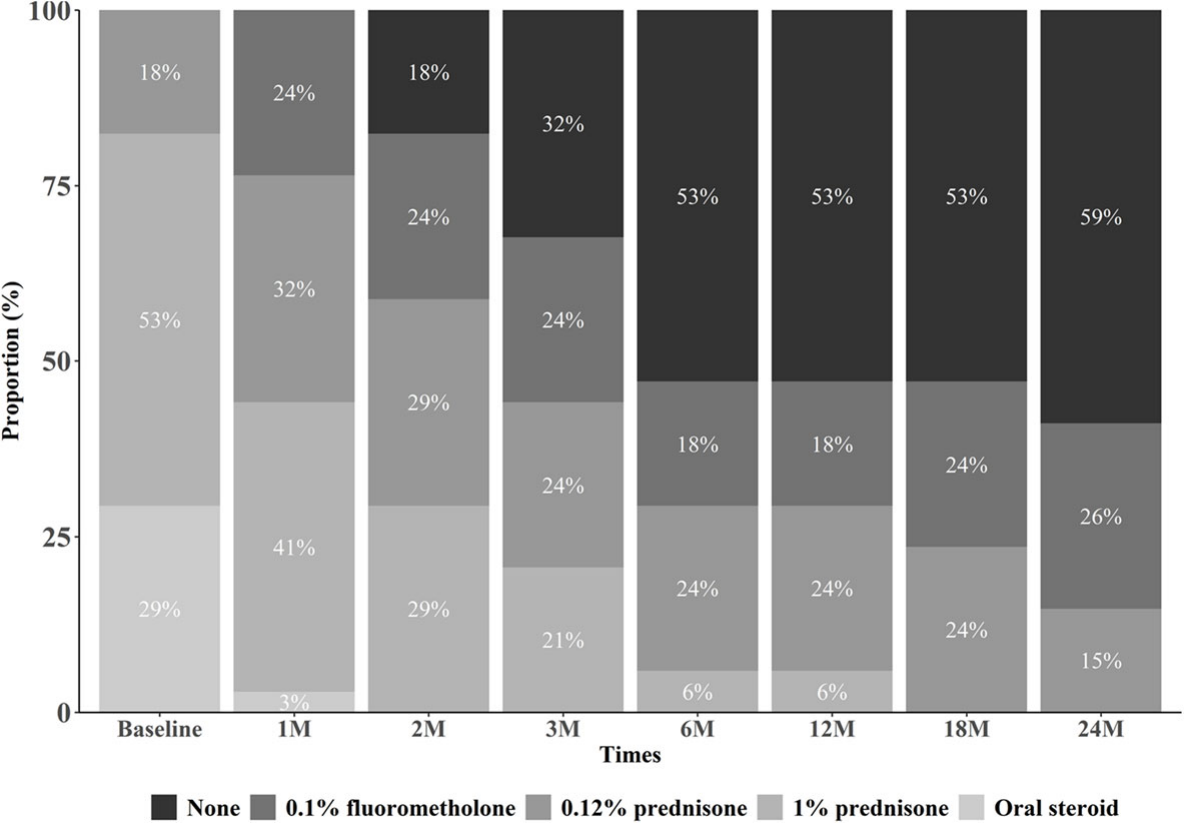
Changes in steroid use in combination with the tacrolimus ointment. It shows that the proportion and need of steroids decreases during the treatment period

During the follow-up, the percentage of eyes receiving adjunctive topical steroid treatment decreased to 82% at 2 months and 47% at 6 months. The percentage of eyes with adjunctive 1% prednisone was 41, 29, and 6% at 1, 2, and 6 months, respectively. The percentage of eyes with adjunctive 0.1% fluorometholone eye drops was 32, 29, and 24% at 1, 2, and 6 months, respectively. More than half of the total patients were treated with tacrolimus alone, successfully weaned off topical steroids at 6 months. (Fig. 4). The steroid score improved significantly from 3.32 ± 0.84 at baseline to 0.58 ± 0.65 at the final follow-up (Table 2).

## Discussion

The current study investigated the long-term safety of topical tacrolimus treatment in pediatric patients with ocular surface inflammation that was refractory to conventional treatment. To our knowledge, this study was conducted with the longest observation period for evaluating the safety and efficacy of topical tacrolimus in pediatric patients.

The largest study till date on topical tacrolimus use in pediatric patients reported its use in 45 patients (mean age: 8.23 ± 2.7 years) of VKC with an average follow-up of eight months. In this study, 89% of patients showed significant improvement and no side effects were reported except that one patient developed subepithelial keratitis [17]. Our study included 34 patients (mean age: 11.2 ± 4.2 years) with follow-up period of 12 months or longer. The average follow-up period reached 23.12 ± 19.07 (range, 12 to 98) months. In addition to VKC, our study included various ocular surface inflammation such as ocular GVHD, OCP, SJS, AKC, and PKC. The results of our analyses showed that majority of patients tolerated the treatment, showed improved symptoms and clinical signs, and required less concurrent steroid therapy. Over 12 months of follow-up, no adverse reaction was noted.

In our study, patients were initially prescribed 0.02% tacrolimus ointment in combination with topical steroids during the active phase because topical tacrolimus requires several weeks to reach the treatment concentration in eyes. On the other hand, topical steroids are fast-acting and promptly relieve symptoms [18–20]. Therefore, topical steroids help resolve inflammation in the cornea and conjunctiva immediately until tacrolimus becomes effective.

The major side effects of topical tacrolimus are eye irritation, blurring, itching, chemosis, transient burning sensation, conjunctival hyperemia, and conjunctival chemosis [5]. Burning sensation, which was the reason behind treatment cessation in our study population, has been documented in previously published reports using higher concentrations (0.01%) but not in those using lower concentrations (0.005%). Hence, it is possible that this side effect is dependent on the drug concentration [5, 21, 22]. In terms of adverse effects, renal toxicity, hyperglycemia, and hypertension have been reported [23]. However, because of the limited amount of tacrolimus used during topical treatment, the risk of these adverse effects is negligible. A study that investigated the blood concentration profile of tacrolimus following topical application, its systemic exposure was reported to be minimal and temporary [24]. There have also been reports of complications such as blood dyscrasias, malignancies and outbreaks of infection including herpes simplex as well as organ damage with the use of tacrolimus. Such complications are thought occur more frequently in children, but no systemic complications were noted in our study. to be greater in children. A possible local adverse effect of topical tacrolimus is an increased predisposition to infections [20]. A study has shown that its long-term usage increases the risk of corneal infections [22]. The prevalence of corneal infections in a large cohort of patients treated with topical tacrolimus was 0.35% [5]. However, no ocular complications were observed during our study.

The study has several limitations. Most patients (80%) were VKC and AKC patients. And because of that, the number of patients who finally maintained treatment for more than 12 months was relatively small. The clinical signs and severity were evaluated by the representative signs common in six disease groups such as conjunctival fibrosis, conjunctival hyperemia, limbal inflammation, and punctate keratopathy. However, these parameters are not enough to evaluate the severity of AKC or VKC. In particular, the Shield’s ulcer and palpebral conjunctival involvement features are essential for evaluating the severity of VKC. This study is not only about AKC or VKC, but it includes six disease groups. So, we could not include the signs that appear only in certain diseases. Further studies with a larger number of subjects and a longer period of follow-up and divided by each ocular surface inflammation would be necessary to verify the safety of topical tacrolimus in pediatric patients with refractory ocular surface inflammation. Despite these limitations, we believe that our long-term observation supported tacrolimus as an effective and safe treatment option in pediatric patients with refractory ocular surface inflammation.

## Conclusions

Long-term treatment of topical tacrolimus 0.02% ointment is safe and effective in refractory ocular surface inflammation in pediatric patients.

## Supplementary Information

**Additional file 1.** Raw data from pediatric patients using topical 0.02% tacrolimus ointment for the treatment of refractory ocular surface inflammation.

## Data Availability

The datasets generated and analyzed during the current study are not publicly available due to protection of the patient’s personal information but are available from the corresponding author on reasonable request.

## Abbreviations

AKC: Atopic keratoconjunctivitis
GVHD: Graft-versus-host disease
OCP: Ocular cicatricial pemphigoid
PKC: Phlyctenular keratoconjunctivitis
SIG: Steroid-induced glaucoma
SJS: Stevens–Johnson syndrome
VKC: Vernal keratoconjunctivitis
